# Early Renal Dysfunction and Reduced Retinal Vascular Density Assessed by Angio-OCT in Hypertensive Patients

**DOI:** 10.3390/biomedicines13051176

**Published:** 2025-05-12

**Authors:** Caterina Carollo, Maria Vadalà, Alessandra Sorce, Emanuele Cirafici, Miriam Bennici, Massimo Castellucci, Vincenza Maria Elena Bonfiglio, Giuseppe Mulè, Giulio Geraci

**Affiliations:** 1 Unit of Nephrology and Dialysis, Hypertension Excellence Centre, Department of Health Promotion, Mother and Child Care, Internal Medicine and Medical Specialties (PROMISE), University of Palermo, 90133 Palermo, Italy; alessandra.sorce@community.unipa.it (A.S.); emanuele.cirafici@community.unipa.it (E.C.); miriam.bennici@community.unipa.it (M.B.); giuseppe.mule@unipa.it (G.M.); 2 Biomedicine, Neuroscience and Advance Diagnostic (BIND) Department, University of Palermo, 90133 Palermo, Italy; maria.vadala@unipa.it (M.V.); massimo.castellucci@policlinico.pa.it (M.C.); vincenzamariaelena.bonfiglio@unipa.it (V.M.E.B.); 3 Biomedicine, Neuroscience and Advanced Diagnostic Department, IEMEST Euro-Mediterranean Institute of Science and Technology, University of Palermo, 90133 Palermo, Italy; 4 Faculty of Medicine and Surgery, Kore University, 94100 Enna, Italy; giulio.geraci@unikore.it

**Keywords:** hypertension, CKD, Angio-OCT, early kidney damage

## Abstract

**Background:** The eye and kidney share embryological, structural, and pathophysiological similarities, suggesting potential interconnections between retinal and renal microvascular changes. Hypertension, a major risk factor for renal impairment, also affects retinal microvasculature. This study investigates the relationship between retinal vascular density, assessed by Optical Coherence Tomography Angiography (OCT-A), and early renal dysfunction in hypertensive patients. **Methods:** A total of 142 hypertensive patients (mean age 47 ± 13 years; 74% male) were enrolled from the Nephrology and Hypertension Unit at the University of Palermo. Retinal vascular density was measured using OCT-A, and renal function was assessed using estimated glomerular filtration rate (eGFR). Clinical and hemodynamic parameters, including 24-h aortic blood pressure, were also analyzed. **Results**: Patients with eGFR < 60 mL/min/1.73 m^2^ exhibited significantly lower retinal vascular densities, particularly in the parafoveal region. Superficial parafoveal density was inversely associated with aortic pulse pressure (*p* = 0.012) and directly correlated with eGFR (*p* = 0.012). Deep parafoveal density was independently associated with eGFR (*p* = 0.001). Multiple linear regression confirmed that lower retinal vascular density was significantly linked to reduced renal function, independent of age and blood pressure. **Conclusions:** Retinal vascular density, particularly in the parafoveal region, is associated with renal function decline in hypertensive patients. These findings suggest that retinal microvascular changes could serve as a non-invasive biomarker for kidney dysfunction, with potential applications in early risk stratification and disease monitoring. Further research is needed to establish causality and clinical utility.

## 1. Introduction

The eye and the kidney share significant embryological, structural, and pathophysiological similarities. Both organs originate from a common developmental pathway and exhibit analogous microvascular architectures, as seen in the structural resemblance between the choroid and the glomeruli. Moreover, they are both highly vascularized and susceptible to similar pathogenic mechanisms, including atherosclerosis, vascular remodeling, endothelial dysfunction, inflammation, oxidative stress, and genetic polymorphisms [1,2,3]. These shared characteristics suggest potential interconnections between renal and retinal pathologies, particularly in the context of systemic vascular diseases such as hypertension [4,5]. The renin–angiotensin–aldosterone system (RAAS) is found in both the kidney and in various ocular tissues. This system is an important regulator of blood volume and systemic vascular resistance [6].

Hypertension is a major risk factor for both renal impairment and retinal microvascular alterations. The evaluation of retinal microvascular parameters may provide insight into early renal dysfunction, offering a non-invasive approach to assess systemic microvascular health. Optical coherence tomography angiography (OCTA) is an advanced imaging modality that allows for detailed visualization and quantification of retinal vascular density. Given the microvascular parallels between the kidney and the retina, OCTA may serve as a useful tool to investigate early microvascular changes in hypertensive patients and their potential correlation with renal function.

The primary aim of this study was to explore the potential relationship between retinal vascular density, as assessed by OCTA, and early functional renal alterations in patients with hypertension. Furthermore, we sought to analyse the associations between retinal vascular density and various clinical and hemodynamic parameters within the same patient cohort. By elucidating these relationships, this study aims to enhance our understanding of the microvascular crosstalk between the eye and the kidney, potentially contributing to the development of novel diagnostic and prognostic tools for hypertensive patients.

## 2. Materials and Methods

The study population was selected from patients attending the he ESH Hypertension Excellence Centre Outpatient clinic of our Nephrology and Hypertension Unit. Written informed consent was obtained from all participants. The study protocol adhered to the principles of the Declaration of Helsinki and was approved by the Local Ethic Committee.

Exclusion criteria for the study included:

- Age <20 years or >70 years.
- Known diabetes or fasting blood glucose > 126 mg/dL.
- Pregnancy.
- Systemic diseases or ocular pathologies such as glaucoma, uveitis, high myopia, or macular degeneration, as well as a history of ophthalmic surgery that could have affected the retinal or choroidal vascular system.
- Nephroparenchymal, renovascular, malignant, or endocrine hypertension, or obstructive sleep apnea syndrome.
- Known hematuria, nephritic diseases and hereditary kidney diseases, absence of certain diagnosis of CKD in the group belonging to eGFR > 60 mL/min/1.73 m^2^, or estimated glomerular filtration rate (eGFR) < 15 mL/min/1.73 m [7].
- Positive history or clinical signs of Heart failure, coronary artery disease, cerebrovascular disease and major non-cardiovascular diseases.
- Any condition preventing reliable blood pressure (BP) measurements using the oscillometric technique (e.g., atrial fibrillation, frequent ectopic beats, second- or third-degree atrioventricular blocks, or an upper arm circumference < 22 cm). Patients with an upper arm circumference > 32 cm were not excluded; instead, an appropriately sized cuff was used to obtain accurate BP measurements.

### 2.1. Study Design

A total of 142 hypertensive patients (mean age 47 ± 13 years; 74% male) were enrolled in the study. All participants underwent the following procedures:

- Routine biochemical assessments;
- 24-h ambulatory blood pressure (BP) monitoring using the oscillometric BP Lab Vasotens device, which also enabled the estimation of central (aortic) blood pressure;
- Optical coherence tomography angiography (Angio-OCT).

Clinical blood pressure was measured as the mean of three consecutive readings, obtained at two-minute intervals, following five minutes of seated rest. Measurements were performed using an automated oscillometric device (WatchBP Office, Microlife AG, Widnau, Switzerland).

For 24-h ambulatory blood pressure monitoring (ABPM), recordings were obtained using the oscillometric BP Lab Vasotens device (BP Lab Vasotens (Petr Telegin LLC, Nizhny Novgorod, Russia)), adhering to the European Society of Hypertension (ESH) guidelines for ABPM acquisition [8]. Blood pressure values were automatically recorded at 15-min intervals during the daytime and every 20 min during the nighttime. A properly sized cuff was applied to the non-dominant arm. Patients were instructed to minimize movement and maintain their usual daily activities during the monitoring period, avoiding excessive physical exertion or significant alterations to their typical routines.

The BPLab oscillometric device also enables the estimation of central (aortic) hemodynamic parameters, including central blood pressure and pulse pressure. The pulse wave profile obtained through ABPM was analyzed using BPLab Vasotens technology, allowing for the calculation of central pulse wave parameters derived from the peripheral pulse wave. During blood pressure measurement, oscillations in the cuff were recorded during gradual deflation. After digitalization, signal processing was performed using a mathematical algorithm based on a generalized transfer function, modified for a specific frequency range. The amplitude of all signals was recorded when the cuff pressure exceeded systolic blood pressure, allowing for the detection of the sphygmographic waveform morphology.

Routine biochemical parameters were determined using standard techniques with an automated analyzer. Low-density lipoprotein (LDL) cholesterol was calculated using the Friedewald formula.

In patients exhibiting proteinuria, even at trace levels, or microalbuminuria based on semi-quantitative dipstick testing, a quantitative 24-h urinary albumin excretion measurement was performed. Urinary albumin levels were assessed using a turbidimetric assay and expressed in milligrams per day (mg/day). Serum creatinine concentrations were determined using a standardized enzymatic method (Creatinine Plus, Roche Diagnostics, Basel, Switzerland). The glomerular filtration rate (GFR) was estimated utilizing the Chronic Kidney Disease Epidemiology Collaboration (CKD-EPI) equation.

The entire study population was categorized into two groups based on a GFR value of >60 or ≤60 mL/min/1.73 m^2^ of body surface area, in accordance with literature data (KDIGO 2012) [9]. According to these guidelines, a GFR below this threshold, when estimated using standard methods, allows for the relatively accurate identification of patients with chronic kidney disease.

Ophthalmological evaluations were performed on all patients, including best-corrected visual acuity assessment using ETDRS (Early Treatment Diabetic Retinopathy Study) charts. Intraocular pressure (IOP) was measured using a Goldmann applanation tonometer. Anterior segment and fundus examinations were performed using slit-lamp biomicroscopy under pharmacologically induced mydriasis with 1% phenylephrine drops, along with a Volk 90D lens.

Optical coherence tomography (OCT) examinations were performed using a Swept-Source OCT (SS-OCT) device (Triton; Topcon Inc., Tokyo, Japan). All imaging procedures were conducted by a single trained operator between 10:00 a.m. and 12:00 p.m., following a standardized scanning protocol. The right eye was examined first in all participants. In cases where scan quality was inadequate, imaging was either repeated or excluded from further analysis. As no significant interocular differences were observed, one eye per participant was randomly selected for analysis using a random number generator. If the quality of the selected eye’s scan was deemed insufficient, the contralateral eye was used instead.

For each eye, the following scanning protocols were used:

- 3D 7x7H
- Macular Radial 6.0
- Angio-OCT 4.5

Retinal thickness, defined as the distance from the inner limiting membrane to the inner surface of the retinal pigment epithelium, and choroidal thickness, measured from the outer border of the retinal pigment epithelium to the scleral interface, were automatically calculated using the OCT device’s mapping software (DRI Triton version 1.04E—1.36.2, Topcon Inc., Tokyo, Japan)) The resulting measurements were presented as mean values with corresponding standard deviations for each of the nine regions delineated by the Early Treatment Diabetic Retinopathy Study (ETDRS) grid.

The ETDRS grid segments the macular and choroidal regions into nine sectors centered on the fovea, composed of three concentric circles with diameters of 1 mm, 3 mm, and 6 mm. The innermost and outermost rings are further subdivided into four quadrants: temporal, nasal, inferior, and superior.

Fovea-centered OCT angiograms (4.5 × 4.5 mm, 320 × 320 pixels) were obtained to assess the superficial and deep retinal capillary networks. In each image, the perimeter of the foveal avascular zone (FAZ) within the superficial vascular plexus was manually outlined by a single operator, and the corresponding area was automatically computed by the OCT software. To reduce measurement variability, the final FAZ area used for analysis was determined by averaging two independent measurements.

Image processing and retinal vascular density analyses were performed using ImageJ software, version 1.49 (National Institutes of Health, Bethesda, MD, USA). Retinal vascular maps were generated by applying an automated thresholding technique. Vascular density was defined as the proportion of the area occupied by blood vessels, identified as pixels exceeding the selected threshold. Quantitative assessments were carried out in two predefined regions of interest (ROIs): the foveal and parafoveal zones. The foveal ROI was defined as a central circle measuring 120 pixels in diameter (equivalent to 1.2 mm), while the parafoveal ROI corresponded to a 91-pixel-wide annular ring surrounding the foveal area.

### 2.2. Statistical Analysis

The Gaussian distribution of continuous variables was assessed using the Kolmogorov-Smirnov test, which confirmed a normal distribution for all variables except urinary albumin excretion and triglycerides, both of which exhibited a positively skewed distribution. These variables were expressed as medians and interquartile ranges and were logarithmically transformed before further statistical analyses. All normally distributed continuous variables were expressed as means and standard deviations, while categorical variables were presented as percentages.

Group differences were evaluated using the independent samples Student’s *t*-test for continuous variables and the chi-square test with Yates’ correction, or Fisher’s exact test when appropriate, for categorical variables. Adjustment for potential confounding factors was performed using analysis of covariance (ANCOVA).

Simple regression analysis and Pearson’s correlation coefficients were used to assess the relationship between retinal vascular densities and other variables. Associations between continuous variables were tested using Pearson’s correlation analysis and simple and multiple linear regression analyses. In particular, to evaluate the independence of the observed relationships, multiple linear regression models were constructed, with each model considering a specific retinal vascular density as the dependent variable and including as independent variables those parameters found to be associated with retinal vascular density in univariate analyses. Continuous variables included as covariates were expressed as variations of one standard deviation (SD).

The null hypothesis was rejected in all two-tailed tests for *p*-values < 0.05.

## 3. Results

Table 1 presents the main clinical, demographic, and anthropometric characteristics of the entire study population, as well as the two groups stratified based on an estimated glomerular filtration rate (eGFR) above or below 60 mL/min/1.73 m^2^.

Patients with chronic kidney disease were found to be older and had higher blood glucose and plasma triglyceride levels compared to individuals with normal renal function. However, the statistical significance for triglyceridemia is borderline.

Table 2 presents the hemodynamic data, including clinical and 24-h ambulatory blood pressure values recorded at the brachial level and estimated at the aortic level, as well as clinical heart rate, for the entire study population and the two groups stratified by eGFR. As shown in the table, the difference between the two groups was found to be significant only for 24-h estimated aortic systolic pressure and aortic pulse pressure.

The table below (Table 3) shows the distribution of patients on antihypertensive medication, along with the various antihypertensive drugs and other medications affecting the cardiovascular system. No statistically significant differences were found between the two groups with different levels of renal function in this regard.

No statistically significant differences were observed in retinal vascular densities when comparing subjects treated with antihypertensive medications to those not treated, as well as when comparing patients on diuretics, alpha-blockers, alpha-beta blockers, AT1 blockers, calcium antagonists, statins, and allopurinol to those not taking these medications.

However, the density of the deep parafoveal plexus was found to be lower in patients treated with platelet aggregation inhibitors (*p* = 0.035) and beta-blockers (*p* = 0.031), while it was higher in patients on ACE inhibitors (*p* = 0.035). The superficial foveal density was lower in the group receiving central sympatholytics (*p* = 0.024).

**Figure 1:**
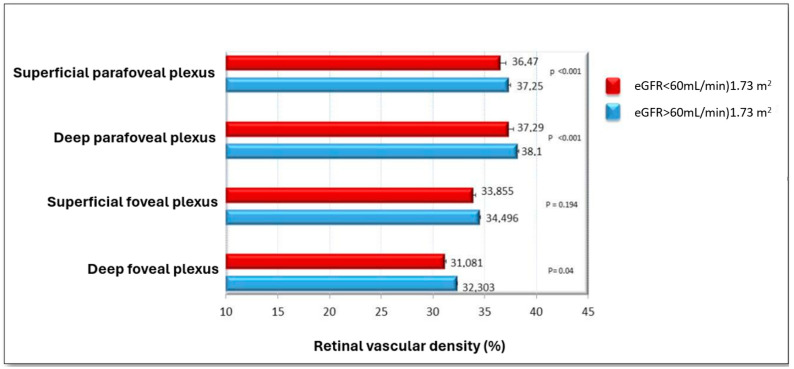
Comparison of retinal vascular density (%) in different retinal plexuses between patients with eGFR < 60 mL/min/1.73 m^2^ (red) and eGFR > 60 mL/min/1.73 m^2^ (blue).

The following graph (Figure 1) illustrates how the retinal vascular density values, both in the parafoveal and foveal plexuses (at both superficial and deep levels), are lower in subjects with a glomerular filtration rate below 60 mL/min/1.73 m^2^ compared to those with normal renal function, with the exception of superficial foveal density, for which statistical significance was not reached.

Furthermore, analysis of covariance (ANCOVA) showed that the differences in retinal vascular density between the two groups, after adjusting for age, blood glucose, triglycerides, and, in the case of the deep parafoveal plexus, after adjusting for medications previously mentioned, remained significant only for the parafoveal region (*p* = 0.003 for the superficial parafoveal plexus and *p* = 0.06 for the deep parafoveal plexus). Table 4 below shows the correlation between retinal vascular density in the parafoveal and foveal plexuses (superficial and deep) and other parameters. Statistical significance was reached in the relationships between the superficial and deep parafoveal plexuses with age, 24-h aortic systolic and pulse pressure, creatinine levels, glomerular filtration rate (GFR) and blood glucose.

The vascular densities of the superficial and deep foveal plexus were significantly correlated only with aortic systolic pressure and glomerular filtration rate. Correlations with age, creatinine levels, blood glucose, and aortic pulse pressure were found to be non-significant.

In the group of patients with positive semi-quantitative analysis of microalbuminuria, comprising 33 subjects, an inverse and significant correlation was observed between the logarithm of urinary albumin excretion and superficial parafoveal vascular density (r = −0.385, *p* = 0.027). Due to the small size of this subgroup, we were unable to perform statistical adjustments to assess whether this relationship is independent of potential confounding factors.

A preliminary multiple linear regression model conducted on the entire study population showed that when retinal vascular densities were considered as dependent variables, the superficial parafoveal density was independently and directly associated with glomerular filtration rate (GFR) and inversely associated with aortic pulse pressure (Table 5 and Table 6). However, statistical significance was not reached regarding age.

The deep parafoveal density (see table below) is independently and directly associated with the glomerular filtration rate and blood glucose levels. Statistical significance was not reached for the estimated aortic pulse pressure.

In a third (Table 7) and fourth (Table 8) multiple linear regression model, superficial and deep foveal vascular densities were independently and directly associated with glomerular filtration rate (GFR), as shown in the tables below.

## 4. Discussion

The results of this study provide valuable insights into the relationship between retinal vascular density and renal function in a cohort of patients with varying degrees of kidney function suggesting that ocular microvascular alterations detected through retinal capillary density analysis using OCTA are clearly associated with established indices of early renal function impairment, such as a mild to moderate reduction in glomerular filtration rate and a slight increase in urinary albumin excretion. It is important to emphasize that the relationship with glomerular filtration rate remains independent of potential confounding factors such as age, sex, blood pressure values, glycemia, and certain medications. Additionally, with regard to parafoveal vascular densities, an independent relationship was observed between increased aortic pulse pressure and retinal microvascular rarefaction.

This observation is also consistent with our previous findings and others research groups in recent literature, where retinal microvascular changes, such as reduced vascular density and altered vascular morphology, have been shown to correlate with renal dysfunction [10,11,12,13].

One of our previous studies, conducted on a smaller population, which also included subjects with primary nephropathies found that patients with CKD had significantly thinner choroidal thicknesses compared to those without CKD, even after adjusting for confounding factors. A significant direct correlation was observed between overall choroidal thickness and glomerular filtration rate (eGFR), and a negative correlation with urinary albumin excretion [14].

In contrast with our findings, the study conducted by Paterson found that inner retinal thinning and retinal microvascular variation were associated with advanced CKD (stages 4 and 5) but not with earlier stage CKD (stage 3), suggesting limited utility as an early biomarker [15]. Interestingly, our findings revealed that the correlation between eGFR and retinal vascular density remained statistically significant even after adjusting for age, glycemia, and triglyceride levels, suggesting that renal function per se plays a direct role in microvascular homeostasis.

The underlying pathophysiological mechanism may involve shared risk factors such as hypertension, diabetes, and atherosclerosis, which affect both renal and retinal vasculature. Furthermore, retinal microvascular changes have been postulated to reflect systemic endothelial dysfunction, which is also implicated in the progression of kidney disease [16]. These results are also similar to those obtained by Chua et al., who found an association between foveal density and glomerular filtration rate similar to what we observed, although this association was statistically significant only for the deep foveal plexus [11]. This suggests that systemic hypertension and arterial stiffness may have differential effects on distinct retinal vascular layers. We hypothesize that a sparser network of retinal capillaries, indicative of microvascular rarefaction, could mirror similar alterations in the renal microcirculation.

Prior studies have reported other structural and functional retinal changes in patients with CKD, including increased vascular tortuosity, narrowed arterioles, and reduced perfusion [17,18].

While previous studies and meta-analysis have emphasized the impact of systemic hypertension on retinal vessel caliber and perfusion [19,20,21], our results suggest that central hemodynamic parameters—rather than peripheral blood pressure measurements, as —may better capture the microvascular burden on the retinal circulation.

In one of our previous studies, no correlation was found between office blood pressure measurements and retinochoroidal thickness or vascular density assessed by OCTA. Therefore, it cannot be excluded that the use of out-of-office blood pressure measurement techniques, such as 24-h ambulatory blood pressure monitoring or self-measurement of blood pressure at home, might reveal a correlation between ophthalmic variables and pressure parameters [10].

Another notable finding in this study is that the estimated glomerular filtration rate (eGFR) failed to show a direct association with retinal thickness. Conversely, eGFR was directly associated with choroidal thickness. The better overall renal function in the chronic kidney disease (CKD) patients enrolled in our investigation (GFR 65 ± 23 mL/min/1.73 m^2^) compared to that observed in the current study and in studies by other authors may explain this inconsistency. It is plausible that the reciprocal relationship between GFR and retinal thickness may change progressively as renal function declines.

The association between aortic pulse pressure and retinal vascular density in our study warrants further discussion.

We found that higher aortic pulse pressure was inversely associated with superficial parafoveal vascular density. This relationship approached statistical significance when considering the deep parafoveal plexus.

The pulsatile nature of central hemodynamics has a detrimental impact on vital organs. In a healthy cardiovascular system, the elasticity of large arteries helps dampen the oscillations in pulse and flow generated by the cyclic systolic contractions of the left ventricle. This buffering effect ensures a continuous blood flow at the microcirculatory level. However, vascular aging, hypertension, and other cardiovascular risk factors contribute to arterial stiffening, reducing the ability of elastic arteries to attenuate these pulsatile forces. Consequently, the microcirculation is increasingly exposed to elevated pulsatile stress. This phenomenon is particularly relevant in organs characterized by high blood flow and low resistance, such as the brain and kidneys, and potentially also the chorioretinal circulation [22].

Aortic pulse pressure, a marker of arterial stiffness, has been shown to independently predict adverse cardiovascular and renal outcomes [23,24,25]. Increased arterial stiffness and elevated pulse pressure can result in microvascular injury and endothelial dysfunction, leading to reduced perfusion in both the kidney and retina. Several studies have documented the correlation between increased arterial stiffness and microvascular abnormalities, particularly in the retina, as seen in conditions such as hypertension and diabetes [26,27]. This may explain the inverse relationship observed in our study, suggesting that aortic pulse pressure could be a key modifiable risk factor for retinal microvascular alterations in patients with CKD. It is plausible that elevated pulse pressure contributes to endothelial dysfunction and capillary dropout, leading to reduced retinal perfusion, as previously proposed in studies on hypertensive retinopathy and cerebral small vessel disease [28,29].

Despite its well-established association with both renal and cardiovascular diseases, age did not significantly correlate with retinal vascular density in our study. This finding contrasts with some studies that have reported an age-related decline in retinal vessel density, particularly in elderly populations [30,31]. However, it is important to note that age-related retinal changes are often subtle and may be overshadowed by more dominant risk factors such as hypertension or diabetes, which were also present in our cohort. Furthermore, the relatively narrow age range of our study population may have limited our ability to detect subtle age-related changes in retinal vascular density. Future studies with larger, age-diverse cohorts may help clarify the role of aging in retinal microvascular changes.

Our study did not find significant differences in retinal vascular density between patients treated with various antihypertensive medications and those who were not. This is in line with a study by Hughes et al. [32], which showed that blood pressure control itself, rather than specific antihypertensive agents, is a key factor influencing retinal microvascular changes. However, we did observe that certain medications, such as platelet aggregation inhibitors, beta-blockers, and ACE inhibitors, were associated with changes in retinal vascular density. Specifically, a lower density was observed in the deep parafoveal plexus in patients treated with platelet aggregation inhibitors and beta-blockers, while ACE inhibitors were associated with higher parafoveal vascular density. These findings align with research suggesting that certain antihypertensive classes may have differential effects on retinal microcirculation, possibly due to their impact on endothelial function and vascular remodeling [33,34]. These observations, albeit preliminary, align with reports suggesting that ACE inhibitors exert protective effects on retinal microcirculation by improving endothelial function and reducing inflammation [35]. Conversely, the potential vasoconstrictive effects of β-blockers on retinal circulation, as previously suggested by studies on ocular perfusion [36] may explain the observed reduction in deep parafoveal density. Further studies with larger cohorts and prospective designs are needed to clarify these pharmacological effects.

Further studies are required to explore how individual pharmacological agents influence retinal vascular health, particularly in patients with CKD, where the optimal antihypertensive treatment regimen may vary based on individual patient characteristics and comorbidities.

While this study provides important insights, several limitations must be considered. First, the relatively small sample size of certain subgroups, particularly those with positive microalbuminuria, limits the generalizability of the results. Additionally, the cross-sectional design of the study does not allow for the establishment of causal relationships. Longitudinal studies are needed to determine whether changes in retinal vascular density precede the onset of CKD or whether retinal vascular changes are a consequence of renal dysfunction. Moreover, the influence of other potential confounders, such as smoking, dietary factors, and genetic predispositions, was not evaluated and could affect the observed relationships.

In conclusion, retinal vascular density is an important marker of microvascular health that correlates with renal function and cardiovascular risk in patients with CKD. This study supports the growing body of evidence suggesting that retinal imaging could serve as a non-invasive tool for monitoring kidney disease and its associated vascular complications. Further research is warranted to explore the potential clinical applications of retinal vascular density measurements in the management of CKD and to elucidate the mechanisms underlying the observed associations.

## 5. Conclusions

This study highlights the significant associations between retinal vascular density and renal function in patients with chronic kidney disease (CKD), providing further evidence that retinal microvascular changes may serve as a non-invasive biomarker for kidney dysfunction. Our findings suggest that reduced retinal vascular density, particularly in the parafoveal region, is linked to a lower glomerular filtration rate (GFR), reflecting the relationship between systemic vascular health and renal function. Our findings have important clinical implications, particularly regarding the potential role of retinal vascular density as a surrogate marker for systemic microvascular health. The retina provides a unique and non-invasive window into systemic microcirculation, and its alterations may reflect early vascular dysfunction in hypertensive and nephropathic patients. Given that retinal microvascular rarefaction appears to be linked to both renal impairment and elevated pulse pressure, retinal imaging techniques such as OCT-A could be integrated into risk stratification models for patients at high cardiovascular risk. Further longitudinal research is needed to explore the causal relationships between retinal vascular changes and kidney function decline, and to investigate the potential clinical applications of retinal imaging in the management of CKD.

## Figures and Tables

**Table 1:** Main clinical, demographic, and anthropometric characteristics of the studied population and in the two subgroups divided by an eGFR threshold of 60 mL/min/1.73 m^2^.

	Total Population (n = 142)	eGFR > 60 mL/min/1.73 m^2^ (n = 118)	eGFR < 60 mL/min/1.73 m^2^ (n = 24)	*p*
Age	47.6 ± 12.8	44.7 ± 12.4	58.4 ± 8.8	<0.001
Male sex%	74%	75%	72%	0.99
BMI (kg/m^2^)	27.9 ± 4.3	28.1 ± 4.4	27.3 ± 4.3	0.491
Waist Circumference (cm)	96.5 ± 11.6	96.5 ± 11.7	96.5 ± 11.2	0.99
Smokers	21.7%	21.1	25%	0.078
GFR-CKD-EPI (mL/min)	89.1 ± 22.7	96.1 ± 17.7	55 ± 7.9	<0.001
(UAE)* (mg/24 h)	70 (33–420)	41.5 (31.5–370.25)	205 (33.1–638)	0.265
Glycemia (mg/dL)	95 + 16	94 ± 16	101 ± 13	0.047
Cholesterol (mg/dL)	194 ± 30	194 ± 32	193 ± 24	0.86
HDL (mg/dL)	49 ± 13	49 ± 12	43 ± 15	0.87
Triglycerides (mg/dL)	110 (78–149)	108.4 (75.8–143)	118 (102–178)	0.052

The data relating to UAE* refer to the 33 patients (20 in the group with GFR > 60 and 12 in the group with GFR < 60) who tested positive for the semi-quantitative evaluation of this parameter and who subsequently carried out the dosage on 24 h urinary collection.

**Table 2:** Main hemodynamic parameters of the studied population and in the two subgroups divided by an eGFR threshold of 60 mL/min/1.73 m^2^.

	Total Population (n = 142)	eGFR > 60 mL/min/1.73 m^2^ (n = 118)	eGFR ≤ 60 mL/min/1.73 m^2^ (n = 24)	*p*
Clinical systolic BP (mmHg)	139 ± 13	139 ± 13	136 ± 15	0.357
Clinical diastolic BP (mmHg)	86 ± 10	87 ± 9	83 ± 10	0.069
Clinical pulse pressure (mmHg)	52 ± 10	52 ± 11	53 ± 11	0.643
Clinical mean pressure (mmHg)	104 ± 10	104 ± 9	101 ± 10	0.104
Clinical heart rate (bpm)	74 ± 11	74 ± 11	76 ± 11	0.253
Mean systolic BP 24 h (mmHg)	131 ± 13	131 ± 12	135 ± 16	0.098
Mean diastolic BP 24 h (mmHg)	82 ± 9	82 ± 9	82 ± 10	0.676
24 h Aortic systolic pressure (mmHg)	125 ± 13	124 ± 13	131 ± 16	0.02
24 h Aortic pulse pressure (mmHg)	43 ± 12	42 ± 12	50 ± 11	0.002

**Table 3:** Distribution of the patients pharmacologically treated for hypertension, of the classes of blood pressure lowering drugs and of other cardiovascular drugs in the whole population and among the subgroups divided by an eGFR threshold of 60 mL/min/1.73 m^2^.

	Total Population (n = 142)	eGFR > 60 mL/min (n = 118)	eGFR < 60 mL/min (n = 24)	*p*
Subjects treated pharmacologically for hypertension (%)	97 (68)	84 (71)	13 (54)	0.622
ACE-inhibitors	17 (12)	12 (10)	5 (20)	0.9999
Sartans	17 (12)	9 (7)	8 (33)	0.581
Diuretics	17 (12)	8 (6)	9 (38)	0.174
Calcium channel blockers	17 (12)	11 (9)	6 (25)	0.579
β-blockers	17 (12)	14 (11)	3 (13)	0.9999
αβ-blockers	17 (12)	14 (11)	3 (13)	0.633
α-blockers	17(12)	14 (11)	3(13)	0.679
Central antiadrenergics	17 (12)	15 (12)	2 (12)	0.760
Statins	17 (12)	11 (9)	6 (25)	0.263
Antiplatelet agents	44 (31)	38 (32)	6 (25)	0.897
Allopurinol	17 (12)	17 (14)	0 (0)	0.393

The number “17” denotes the total number of patients receiving at least one of the medications listed; subgroups corresponding to each drug class may overlap but are not identical. Although treatment duration is not presented in the table for conciseness, patients had been maintained on stable antihypertensive therapy for a minimum of six months prior to enrollment.

**Table 4:** Correlations between Retinal Vascular Density and other clinical parameters.

		Age	24 h Aortic SBP	Aortic PP	Creatinine	eGFR	Glycemia
Vascular density– Superficial parafoveal plexus	r =	−0.287	−0.226	−0.303	−0.320	0.339	−0.22
	*p* =	<0.001	0.008	<0.001	<0.001	<0.001	0.001
Vascular density– Deep parafoveal plexus	r =	−0.292	−0.197	−0.262	−0.350	0.382	−0.286
	*p* =	<0.001	0.02	0.002	<0.001	<0.001	0.001
Vascular density– Superficial foveal plexus	r =	NS	−0.183	NS	NS	0.245	NS
	*p* =	NS	0.03	NS	NS	0.003	NS
Vascular density– Deep foveal plexus	r =	NS	−0.197	NS	NS	0.233	NS
	*p* =	NS	0.02	NS	NS	0.007	NS

**Table 5:** Multiple linear regression analysis for superficial parafoveal retinal vascular density.

Dependent Variable: Superficial Parafoveal Retinal Vascular Density	B*	95% C.I. for B		β^	*p*
Covariates		Lower	Upper		
Variations of one SD for glomerular filtration rate	0.21	0.05	0.38	0.22	0.012
Variations of one SD for estimated aortic pulse pressure	−0.20	−0.36	−0.04	−0.2	0.012
Variations of one SD for Age	−0.15	−0.31	0.01	−0.16	0.07
Constant	37.07	36.92	37.21		0.000

Sex and serum glucose were excluded from the model as they did not contribute significantly to the prediction of retinal vascular density. B*: unstandardized regression coefficient. β^: standardized regression coefficient.

**Table 6:** Multiple linear regression analysis for deep parafoveal retinal vascular density.

Dependent Variable: Deep Parafoveal Retinal Vascular Density	B*	95% C.I. for B		β^	*p*
Covariates		Lower	Upper		
Variations of one SD for estimated glomerular filtration rate	0.352	0.169	0.536	0.303	0.001
Variations of one SD for estimated aortic pulse pressure	−0.178	−0.36	0.007	−0.149	0.06
Variations of one SD for glycemia	−0.242	−0.42	−0.62	−0.206	0.009
Constant	37.91	37.74	38.09		0.000

Other variables that did not reach statistical significance and were excluded from the model include sex, age, and treatment with beta-blockers, ACE inhibitors, and antiplatelet agents. B*: unstandardized regression coefficient. β^: standardized regression coefficient.

**Table 7:** Multiple linear regression analysis for superficial foveal retinal vascular density.

Dependent Variable: Superficial Foveal Retinal Vascular Density	B*	95% C.I. for B		β^	*p*
Covariates		Lower	Upper		
Variations of one SD for estimated glomerular filtration rate	0.302	0.04	0.56	0.187	0.024
Constant	33.93	33.41	34.44		0.000

Other variables that did not reach statistical significance and were excluded from the model include sex, age, aortic PP, glycemia and treatment with central antiadrenergic agents. B*: unstandardized regression coefficient. β^: standardized regression coefficient.

**Table 8:** Multiple linear regression analysis for deep foveal retinal vascular density.

Dependent Variable: Deep Foveal Retinal Vascular Density	B*	95%C.I. for B		β^	*p*
Covariates		Lower	Upper		
Variations of one SD for estimated glomerular filtration rate	0.34	0.21	0.68	0.176	0.037
Constant	32.12	31.79	32.45		0.000

Other variables that did not reach statistical significance and were excluded from the model include sex, age, aortic PP, glycemia and treatment with central antiadrenergic agents. B*: unstandardized regression coefficient. β^: standardized regression coefficient.

## Data Availability

Data are available on reasonable request.
